# Landslide Hazard Identification and Prediction in Complex Mountainous Areas Using Ascending and Descending Orbits InSAR Technology

**DOI:** 10.3390/s26082455

**Published:** 2026-04-16

**Authors:** Wenmiao Zhao, Pengfei Cong, Xu Ma, Mingxuan Yi, Chong Liu, Jichao Gao, Yan Zhang

**Affiliations:** 1 Langfang Integrated Natural Resources Survey Center, China Geological Survey, Langfang 065000, China; zhaowenmiao1112@mail.cgs.gov.cn (W.Z.);; 2 Innovation Base of Natural Resources Change Observation and Capital Monitoring in the Northern Haihe River Basin, China Society of Territorial Economists, Langfang 065000, China; 3 Department of Geologic Engineering, Qinghai University, Xining 810016, China

**Keywords:** landslide identification, deformation prediction, SBAS-InSAR, LSTM

## Abstract

Time-series InSAR is an important means for early identification and monitoring of landslides. However, in complex mountainous areas, it still faces challenges such as significant geometric distortions and complicated deformation mechanisms. To address these issues, this paper proposes a landslide identification and prediction framework that integrates ascending and descending orbits InSAR observations with physics-guided deep learning. Taking Yangbi County, Yunnan Province, as a case study, we combined ascending and descending Sentinel-1A data and employed the SBAS-InSAR method to identify potential landslides, detecting a total of 41 hazardous sites. The cumulative displacement time series of typical landslides were further extracted along the slope aspect to more realistically reflect landslide movement characteristics. On this basis, wavelet decomposition was introduced to separate the displacement series into trend and periodic components. Gray relational analysis was then used to select influencing factors such as precipitation and temperature, and a stepwise prediction model based on LSTM (WT-LSTM) was constructed. The results indicate that the model achieves significantly higher prediction accuracy at characteristic points of the representative landslide (RMSE = 1.16–2.19 mm) compared to standalone LSTM and SVR models. These findings demonstrate its effectiveness and potential applicability in landslide deformation monitoring and prediction in complex mountainous areas, while also providing a useful reference for landslide risk early warning.

## 1. Introduction

The mountainous areas of Yunnan Province in China are highly prone to landslide disasters, primarily due to the interaction among their unique geological structure, climatic conditions, and topography [1,2]. Located on the southeastern margin of the Qinghai–Tibet Plateau, this region experiences active tectonic movements and well-developed faults, providing abundant material sources for landslide formation [3]. Climatologically, the area is influenced by the southwest monsoon, which brings concentrated and intense rainfall—the most direct external trigger for landslides [4,5]. In addition, strong seismic activities (such as the recent Yangbi and Ludian earthquakes) not only directly trigger landslides but also profoundly alter the stress state of slopes and the mechanical properties of rock and soil, forming a coupled earthquake–rainfall hazard chain effect [6,7]. This results in landslides in this region often exhibiting characteristics of clustering, hysteresis, and repeated activity [8,9]. Landslide disasters not only cause substantial economic losses but also lead to severe casualties [10,11]. Major landslide events, such as the Maoxian landslide in Sichuan Province [12,13] and the Zhenxiong County landslide in Yunnan Province [14,15], have been widely reported, posing a serious threat to the safety of local residents’ lives and property [16]. Therefore, early identification, monitoring, and accurate prediction of potential landslides are crucial, as they facilitate early warning of major landslide disasters and effectively reduce casualties and property losses.

However, landslide monitoring in mountainous areas still faces significant challenges. Traditional ground-based measurement methods and Global Navigation Satellite System (GNSS) techniques suffer from low efficiency and high costs, making it difficult to meet the requirements for long-term, large-scale landslide disaster monitoring and identification [10,17]. In this context, the development of Interferometric Synthetic Aperture Radar (InSAR) has provided a more efficient solution. With advantages such as wide coverage, high monitoring accuracy, and short revisit cycles, InSAR has gradually become an important tool for landslide monitoring [18,19,20]. In recent years, with the continuous improvement in SAR satellite systems, the increasing availability of SAR imagery has laid a solid foundation for the advancement of time-series InSAR techniques [21,22,23]. Among them, Differential InSAR (D-InSAR) [24], Persistent Scatterer InSAR (PS-InSAR) [25], and Small Baseline InSAR (SBAS-InSAR) [26] are the most widely applied. D-InSAR is suitable for capturing significant surface deformation over short time periods and large areas, typically providing deformation maps in centimeters, and is often used for the initial identification of earthquakes, volcanic activities, or rapid landslides [27]. PS-InSAR typically relies on permanent scatterers such as buildings or exposed bedrock to achieve high-precision deformation monitoring [28]. In contrast, SBAS-InSAR is more applicable to areas with time-varying scatterers, such as vegetation-covered regions, farmlands, or natural slopes, and can reliably obtain large-scale, continuous surface deformation time series [29,30]. Therefore, SBAS-InSAR is often prioritized for landslide monitoring in complex mountainous areas with dense vegetation, such as Yunnan Province, due to its superior coherence preservation capability.

However, InSAR is inherently limited by its imaging geometry. Single-track SAR data can only capture deformation along the satellite Line of Sight (LOS) direction. In rugged mountainous terrain, geometric distortions such as foreshortening, layover, and shadowing further lead to displacement information bias or loss [31,32]. To address these limitations, researchers commonly integrate multi-track SAR data or introduce prior constraints to enhance the reliability of monitoring results [33,34]. Given the difficulty in acquiring multi-track data, incorporating prior information consistent with landslide kinematic characteristics has become a more commonly adopted approach [35,36]. For instance, Samsonov et al. (2020) proposed a multi-dimensional small baseline subset (MSBAS) technique capable of retrieving three-dimensional time-series deformation of large landslides or glaciers [37]. Jia et al. (2022) combined least squares and Kalman filtering to estimate three-dimensional surface displacements of landslides using dual-track data, thereby revealing their spatial movement patterns more intuitively [38]. Chen et al. (2025) successfully reconstructed the three-dimensional deformation field of the Lanuza landslide by integrating a strain model with a Bayesian inversion framework (SM-BIF), achieving results superior to traditional pixel offset tracking (POT) [39]. These studies demonstrate that incorporating appropriate prior constraints can significantly improve the accuracy of landslide deformation monitoring in complex terrain.

High-precision deformation monitoring data serve as the foundation for landslide early warning, while achieving reliable trend prediction based on time-series deformation information represents another key challenge in current research. Traditional landslide deformation prediction primarily relies on ground-based monitoring techniques such as leveling and GNSS [16]. Although these methods offer high accuracy, they are difficult to implement over long time periods and large spatial scales, and data acquisition is particularly challenging in remote mountainous areas [40,41]. In contrast, InSAR can acquire historical landslide deformation time series with relatively high monitoring accuracy, thereby providing more substantial data support for predictive models [42].

With the rapid advancement of deep learning technologies, landslide deformation prediction methods have evolved from early statistical analyses to the current AI-driven stage, significantly enhancing both prediction accuracy and application scope [43,44,45]. However, landslide deformation is influenced by multiple coupled factors, and single prediction models often struggle to achieve reliable results. Consequently, integrating machine learning methods with signal decomposition algorithms has emerged as an important approach to improving landslide displacement prediction performance [46,47]. Guo et al. (2020) combined wavelet analysis (WA) with a back-propagation neural network (BPNN) to predict GNSS monitoring data from the Outang landslide after decomposition [48]. Liu et al. (2021) employed variational mode decomposition (VMD) and a periodic neural network (PNN) to model and accurately predict the step-like displacement characteristics of the Baishuihe landslide [49]. To address the gradient explosion problem in long time series, Nava et al. (2023) [50] introduced the Long Short-Term Memory (LSTM) network [51], demonstrating its reliability for landslides with low displacement amplitudes. Zhou et al. (2024) extracted landslide deformation time series from multi-temporal InSAR data and combined signal decomposition with machine learning methods to predict trend and periodic displacements separately, achieving efficient large-scale landslide deformation prediction [44]. Furthermore, Chang et al. (2025) proposed a three-dimensional landslide displacement prediction strategy that integrates InSAR LOS observations with GNSS data to reconstruct three-dimensional displacement time series, and effectively predicted the deformation trend of the Xinpu landslide using multiple machine learning algorithms including Random Forest (RF), Support Vector Machine (SVM), LSTM, and Gated Recurrent Unit (GRU) [52]. These studies demonstrate that the landslide displacement prediction paradigm combining signal decomposition and machine learning has shown considerable promise. However, for landslides under different topographic and environmental conditions, systematic consideration of key driving factors remains essential to further improve prediction accuracy and reliability.

Although current research has made considerable progress in landslide monitoring, identification, and displacement prediction, most studies focus on individual components. Systematic investigations that integrate regional landslide hazard identification, slope aspect deformation extraction, and displacement prediction remain relatively scarce, and a mature technical framework applicable to the full-process risk assessment of landslides in complex mountainous areas has yet to be established. To address this gap, this paper proposes a landslide identification and prediction framework that combines ascending and descending orbit InSAR observations with a physics-guided deep learning model. Focusing on the geometric distortion and climatic characteristics of landslide monitoring in mountainous Yunnan, the Yangbi County area was selected as the study site. First, ascending and descending Sentinel-1A data were jointly employed with the SBAS-InSAR method to identify potential landslides, and slope aspect cumulative displacements were derived based on landslide deformation characteristics. Subsequently, wavelet decomposition was introduced to decompose the displacement time series, and influencing factors were selected to construct a stepwise LSTM prediction model integrated with factor analysis, enabling reliable landslide displacement prediction. The superiority of the proposed approach was validated through comparisons with multiple models. This study provides a methodological reference for the early identification and warning of landslide disaster risks in complex mountainous environments.

## 2. Study Area and Data

**Figure 1:**
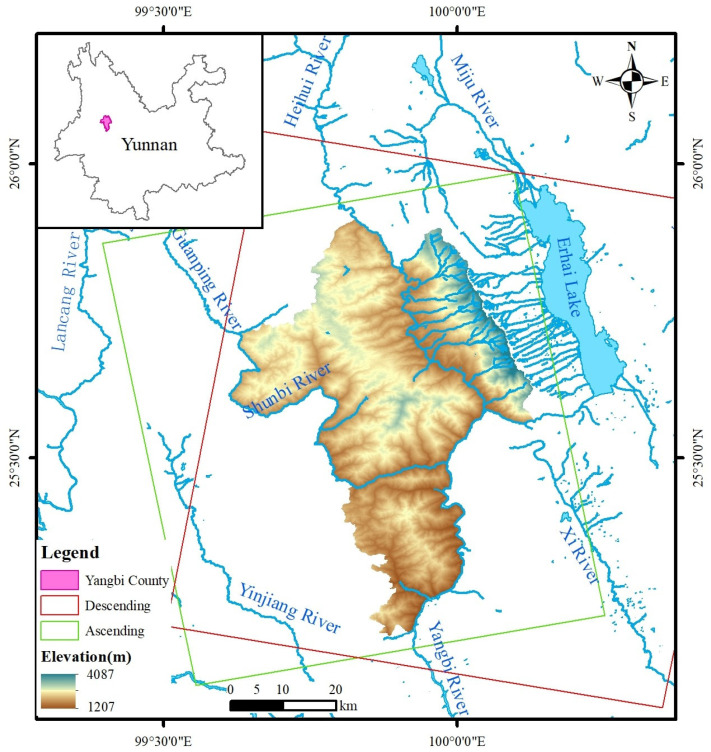
Study area and SAR image coverage.

The study area of this paper is Yangbi County, Dali City, Yunnan Province (99°36′ E–100°07′ E, 25°12′ N–25°54′ N). The county is situated within the Yunling Mountains and is characterized by alpine canyon terrain, with mountainous areas accounting for over 97% of its total land area and flat ground covering less than 3% (Figure 1). The region features a dense river network with well-developed water systems, mostly branching in dendritic patterns through the mountains. The Yangbi River and Shunbi River flow from north to south across the entire county [53]. Yangbi County has a subtropical and temperate plateau monsoon climate, with rainfall concentrated in summer. Influenced by topography and wind direction, mountainous areas receive relatively high precipitation, making them prone to localized landslides and debris flows [54].

Yangbi County is situated between two distinct tectonic units and experiences frequent neotectonic activity. The eastern Honghe Fault Zone and the northern Qiaowei Fault Zone, in particular, are tectonically active and experience frequent seismicity. On 21 May 2021, a 6.4-magnitude earthquake struck Yangbi County, characterized by a deep seismic source (focal depth of approximately 8 km) and numerous aftershocks, resulting in extensive building damage and casualties [55]. Consequently, Yangbi County exhibits high overall susceptibility to geological hazards, among which landslides are particularly prominent. According to the 2022 Yunnan Province Yangbi County Geological Hazard Inventory, which was obtained from the local management authority, there are over 300 geological hazard sites within the county, including 232 landslides, accounting for approximately 69% of the total. This indicates that landslide hazards in Yangbi County are well developed and spatially concentrated, providing favorable conditions for systematic monitoring and prediction research. The area thus serves as an important reference for understanding landslide activity patterns in mountainous regions under similar geological and climatic settings and for developing regional hazard early warning methodologies.

This study primarily collected Sentinel-1A ascending and descending orbit data from October 2017 to April 2023 for landslide hazard identification in the study area, comprising 153 ascending and 127 descending images. The cropped coverage is shown in Figure 1, with detailed parameters provided in Table 1. In addition, precise orbit determination (POD) data and 30 m resolution SRTM DEM data were simultaneously acquired to support time-series InSAR processing [56,57]. Meteorological data from the National Centers for Environmental Information (NCEI) were also collected for subsequent landslide triggering factor analysis and deformation prediction modeling.

## 3. Methods

**Figure 2:**
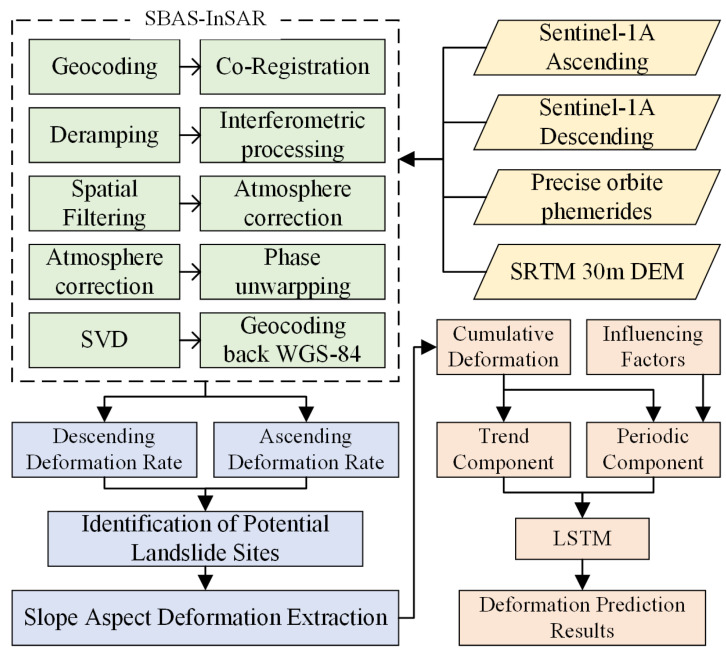
SBAS-InSAR Data Processing and Deformation Prediction Workflow.

This study systematically analyzes landslide hazards from two core components: hazard identification and deformation prediction. First, based on SBAS-InSAR, ascending and descending orbit SAR data are employed to identify potential landslides in the study area and derive surface deformation rate information. Subsequently, typical landslides are selected to extract their slope aspect time-series deformation characteristics, and a prediction model is constructed to quantitatively estimate future deformation trends. Figure 2 illustrates the complete workflow, from data preprocessing and deformation information extraction to predictive modeling.

### 3.1. InSAR Deformation Monitoring

**Figure 3:**
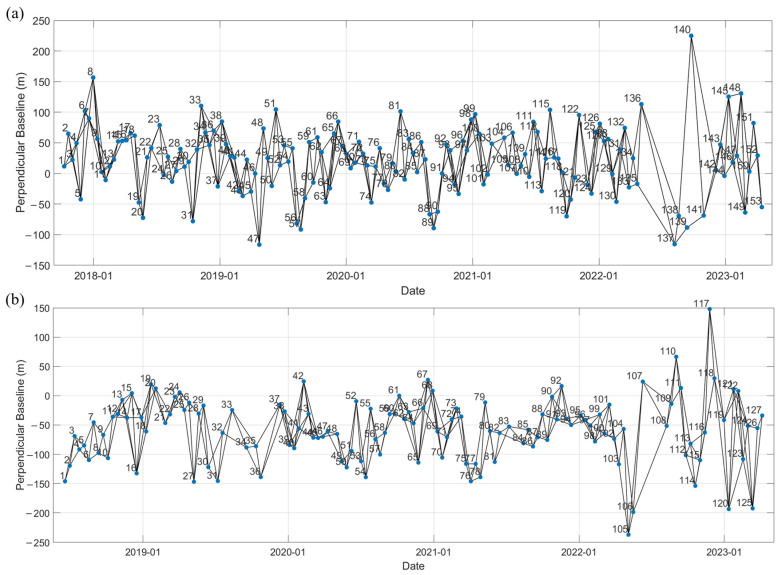
Temporal and spatial baseline distribution. (**a**) Ascending orbit. (**b**) Descending orbit.

This study employs the SBAS-InSAR technique to retrieve LOS surface deformation from ascending and descending Sentinel-1A data in Yangbi County [26]. SBAS-InSAR data processing primarily consists of three stages: data preprocessing, image differential interferometry, and deformation estimation. First, the acquired ascending and descending orbit data covering the study area were separately geocoded and co-registered. Subsequently, a maximum temporal baseline of 36 days and a spatial baseline threshold of 300 m were selected. Due to missing Sentinel-1A images in the study area between June and August 2022, additional pairing was performed, resulting in maximum temporal baselines of 108 days for the ascending orbit and 84 days for the descending orbit. Differential interferometric processing was then conducted. Finally, 357 interferometric pairs were obtained from the ascending data, and 290 pairs from the descending data (Figure 3).

Goldstein filtering [58] was applied to remove topographic and flat-earth phase components from each interferogram, thereby improving the signal-to-noise ratio. Phase unwrapping was then performed using the minimum cost flow (MCF) method [59]. The unwrapped phase $\left(\varphi_{unw}\right)$ primarily comprises four components, as expressed in Equation (1). Linear regression was employed to remove residual topographic phase $\left(\varphi_{res}\right)$, while spatiotemporal filtering was applied to mitigate atmospheric phase $\left(\varphi_{atm}\right)$ [60]. Finally, singular value decomposition (SVD) was used to estimate the deformation phase component $\left(\varphi_{disp}\right)$. After geocoding, LOS deformation rates for both ascending and descending orbits were obtained, and cumulative displacements were further calculated.

(1) $$\varphi_{unw}=\varphi_{res}+\varphi_{atm}+\varphi_{disp}+\varphi_{noise}$$

Since the actual deformation displacement of landslide hazards primarily occurs along the slope–aspect direction of the sliding body, transforming LOS deformation into slope aspect displacement can more realistically reflect the sliding characteristics and movement mechanisms of landslides [61]. The LOS deformation obtained from ascending and descending orbit SAR data can be decomposed into slope aspect displacement through geometric relationships, with the transformation model expressed in Equations (2) and (3). Due to temporal differences in the acquisition of ascending and descending orbit data, time registration is required prior to joint calculation. In this study, taking the acquisition time of descending orbit data as the reference, cubic interpolation [62] was applied to resample the ascending LOS deformation series to align with the descending temporal baseline. Missing sequences in both ascending and descending deformation series were supplemented through interpolation, resulting in ascending and descending deformation time series with identical temporal coverage and equal time intervals (12 days).

(2) $$\left\{\begin{array}{c} D_{\mathrm{as}}=cos\theta_{\mathrm{as}}D_{U}-sin\theta_{\mathrm{as}}cos\varphi_{\mathrm{as}}D_{E}\\ D_{de}=cos\theta_{de}D_{U}-sin\theta_{de}cos\varphi_{de}D_{E} \end{array}\right.$$

(3) $$D_{Slope}=D_{U}/\sin\alpha +D_{E}/\cos\alpha \sin(\beta -\pi /2)$$

Here, $D_{Slope}$ represents the slope aspect displacement, $D_{as}$ and $D_{de}$ are the LOS displacement from ascending and descending orbits, respectively, $D_{E}$ and $D_{U}$ denote the east–west and vertical deformations, respectively. $\theta_{as}$ and $\theta_{de}$ represent the incidence angles of the ascending and descending satellites, respectively. α and β represent the local slope gradient and slope aspect of the landslide, respectively.

### 3.2. Prediction Analysis

The prediction analysis in this study adopts a strategy that combines deformation decomposition with deep learning models. The time-series slope aspect displacements of potential landslide sites obtained through InSAR are decomposed into trend and periodic components. Correlation analysis is then conducted between the periodic component and relevant influencing factors to explore the most suitable landslide prediction approach for complex terrain conditions.

#### 3.2.1. Decomposition of Landslide Deformation Time Series

The occurrence and evolution of landslide deformation are influenced by multiple factors, primarily categorized as internal dynamic forces and external factors, resulting in non-stationary deformation time series. The total landslide displacement W(t) is generally decomposed into three components: the trend component, Q(t) the periodic component, Z(t) and the random component S(t) (Equation (4)). The trend component predominantly represents the overall tendency of landslide deformation, while the periodic and random components reflect deformations induced by seasonal natural variations or external uncertain factors. Given the high uncertainty of the random component and its relatively minor contribution to overall landslide evolution, this study consolidates the periodic and random components, focusing solely on the analysis of trend and periodic deformation components.

(4) W(t)=Q(t)+Z(t)+S(t)

In this study, the continuous landslide deformation time series were decomposed using the Wavelet Transform (WT) method [63]. Through extensive experimentation and comparison, a four-level decomposition scheme employing the Daubechies wavelet basis function was established. High-frequency and low-frequency signals were extracted from the original deformation for each characteristic point, where the low-frequency signals represent the trend component of landslide deformation, and the high-frequency signals correspond to the periodic component.

#### 3.2.2. Prediction Model and Methodology

**Figure 4:**
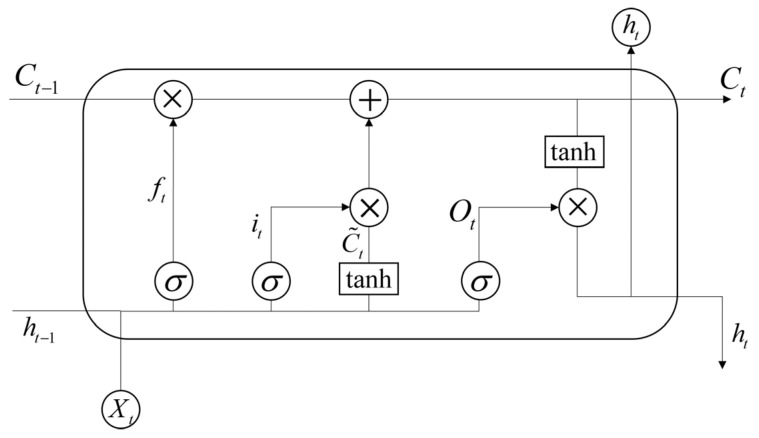
The structure of the LSTM network. $h_{t-1}$ is the output from the previous time step; $X_{t}$ is the input information; σ is the sigmoid function; $h_{t}$ is obtained from the output gate $o_{t}$ and cell state $C_{t}$; the cell state $C_{t}$ is calculated from the input gate $i_{t}$ and hidden gate $f_{t}$; ${\tilde{C}}_{t}$ represents the cell state update value, and finally, the output for the next layer $h_{t}$ is calculated from the output gate $o_{t}$ and cell state $C_{t}$.

To investigate the future deformation trends of landslides in the study area, characteristic points of typical landslides were selected for displacement prediction analysis. This study constructs a hybrid prediction model integrating Wavelet Transform (WT) and Long Short-Term Memory (LSTM) networks (WT-LSTM). As an improved architecture of Recurrent Neural Networks (RNNs), LSTM effectively mitigates the gradient vanishing and explosion problems in long-term dependencies through the introduction of input gates, forget gates, output gates, and cell state mechanisms, while better capturing dynamic features in time-series data [57]. The LSTM structure is illustrated in Figure 4.

The LSTM model adjusts inputs and outputs through sigmoid and tanh activation functions, enabling selective updating of historical data, facilitating the establishment of time-series models and the learning of long-term dependencies, while efficiently transmitting useful information. Consequently, it is well suited for landslide deformation prediction.

Based on the trend and periodic components decomposed via wavelet transform, Gray Relational Analysis (GRA) [64] was employed to identify influencing factors significantly correlated with the periodic deformation component. Factors exhibiting higher correlation were selected as input variables for periodic component prediction. For model training, the dataset was first divided into training and testing sets in chronological order with a ratio of 9:1 to avoid potential information leakage. Interpolation and wavelet decomposition were then performed separately on the training and testing sets, with decomposition parameters derived from the training data and consistently applied to the testing set. Subsequently, separate LSTM models were constructed for training the trend component and the periodic component (integrated with influencing factors). Finally, the predicted total landslide displacement was obtained by summing the predicted values of the trend and periodic components.

## 4. Results and Analysis

### 4.1. Landslide Deformation Monitoring

#### 4.1.1. Identification of Landslide Hazards

**Figure 5:**
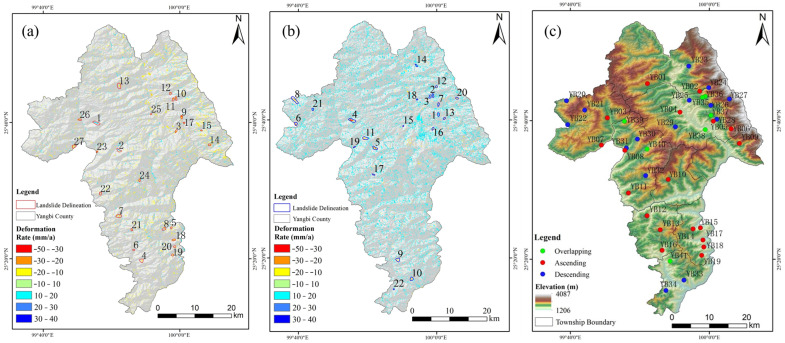
Delineation results of potential landslide points in Yangbi County. (**a**) Deformation rate map of ascending orbit. (**b**) Deformation rate map of descending orbit. (**c**) Distribution map of potential landslide points.

LOS deformation rates for ascending and descending Sentinel-1A data in Yangbi County were obtained using the SBAS-InSAR technique. The results indicate that most areas of Yangbi County exhibit relatively stable surface deformation, with absolute deformation rates generally below 10 mm/a. Therefore, ±10 mm/a was selected as the threshold for surface deformation, and landslide hazards were delineated primarily in areas where deformation rates exceeded the [−10, 10] mm/a range [65,66]. The maximum LOS deformation rates within the study area reached −46 mm/a and −48 mm/a for ascending and descending orbits, respectively (Figure 5a,b). Based on the deformation zones, manual interpretation was conducted using high-resolution optical imagery (from Google Earth and Gaofen-2) to exclude surface anomalies caused by non-landslide factors such as agricultural activities and construction. The slope morphology derived from the DEM was considered, and geological maps and hydrological data were consulted to assess the spatial context of the deformation anomalies, such as proximity to fault zones and rivers. By integrating these auxiliary datasets with the InSAR deformation results, a total of 43 potential landslide hazard sites were preliminarily delineated.

Subsequently, field investigations were conducted on the preliminarily delineated potential landslide sites to observe characteristics such as slope condition, scale, surface cover, and building damage. Among these, 41 sites exhibited environmental characteristics indicative of landslide development, including sparse or exposed surface vegetation, loose soil, fractured bedrock, and visible building damage [67,68]. Therefore, combined with the InSAR monitoring results, these 41 sites were comprehensively identified as potential landslide hazards (Table 2). Among these, 27 sites were identified from ascending orbit data, 22 from descending orbit data, and 8 sites were identified by both orbits (Figure 5c). The identification results were compared with the geological hazard inventory of Yangbi County, revealing that 13 and 10 landslide hazards identified by the ascending and descending orbits, respectively, coincided with the inventory locations, while 14 and 12 sites were newly identified hazards. The ascending and descending orbit results mutually supplemented and validated each other, thereby enhancing the reliability of potential landslide detection.

According to the identification results of landslide hazards derived from combined ascending and descending orbit InSAR data, landslide hazards in Yangbi County are widely distributed, exhibiting a significant spatial correlation with the topographic and tectonic characteristics of the county. Potential landslide sites are dispersedly distributed across the county, with localized clustering patterns. Specifically, concentrated landslide hazard areas are primarily located in Cangshanxi Town in the northeastern part of Yangbi County. Secondary concentrations of potential landslide sites are mainly distributed along the Yangbi River system of the Lancang River basin and near the eastern seismic fault zone, predominantly involving three townships: Fuheng Township, Taiping Township, and Wachang Township.

#### 4.1.2. Typical Landslide Hazard Deformation Analysis and Slope-Oriented Deformation Extraction

Based on the preliminary identification results of landslide hazard points in Yangbi County, combined with field verification, several landslide hazard points with relatively large deformation rates and high risk levels were discovered. Among these, the Zhazishu landslide hazard (YB40) exhibits significant InSAR deformation, obvious deformation characteristics in optical imagery, and concentrated threat targets; therefore, it was selected as a typical case for comprehensive analysis. The Zhazishu landslide hazard is located in Zhazishu Village, Taiping Township, Yangbi County (99°51′4.24″ E, 25°35′54.88″ N), with slope top elevation approximately 2478 m, slope bottom elevation approximately 2025 m, slope length about 1383 m, area about 0.66 km^2^, volume about 281 × 10^4^ m^3^, classifying as a large-scale landslide. From the InSAR deformation monitoring results, the Zhazishu landslide hazard shows significant deformation in both ascending and descending orbits. The deformation range of the descending orbit is −49 to −10 mm/a, while that of the ascending orbit is 10 to 45 mm/a. The deformation rates of both orbits are concentrated between −45 and −30 mm/a, and the deformation extents and magnitudes are generally consistent. Due to the opposite direction between the LOS direction of the ascending orbit and the main sliding direction of the landslide, the deformation rate results are represented as positive values.

**Figure 6:**
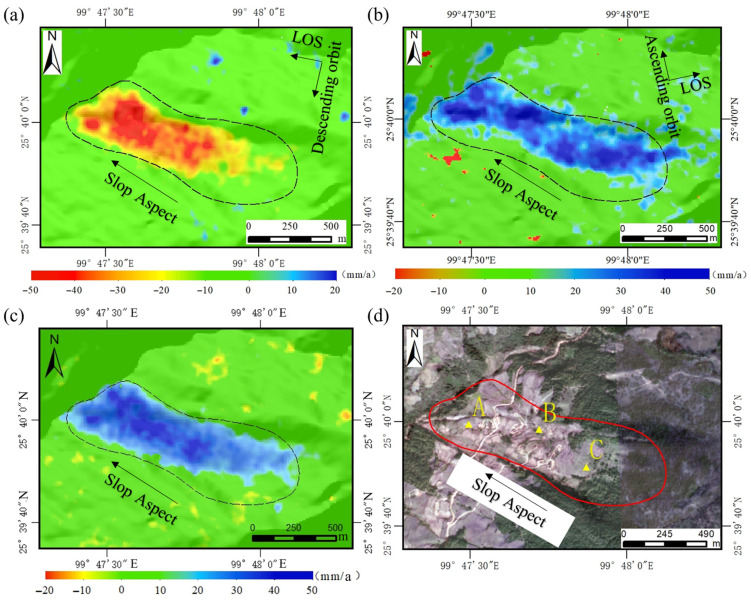
Deformation rate maps of Zhazishu landslide: (**a**) descending orbit deformation rate map, (**b**) ascending orbit deformation rate map, (**c**) slope-oriented deformation rate map, and (**d**) schematic diagram of characteristic points A, B, and C.

**Figure 7:**
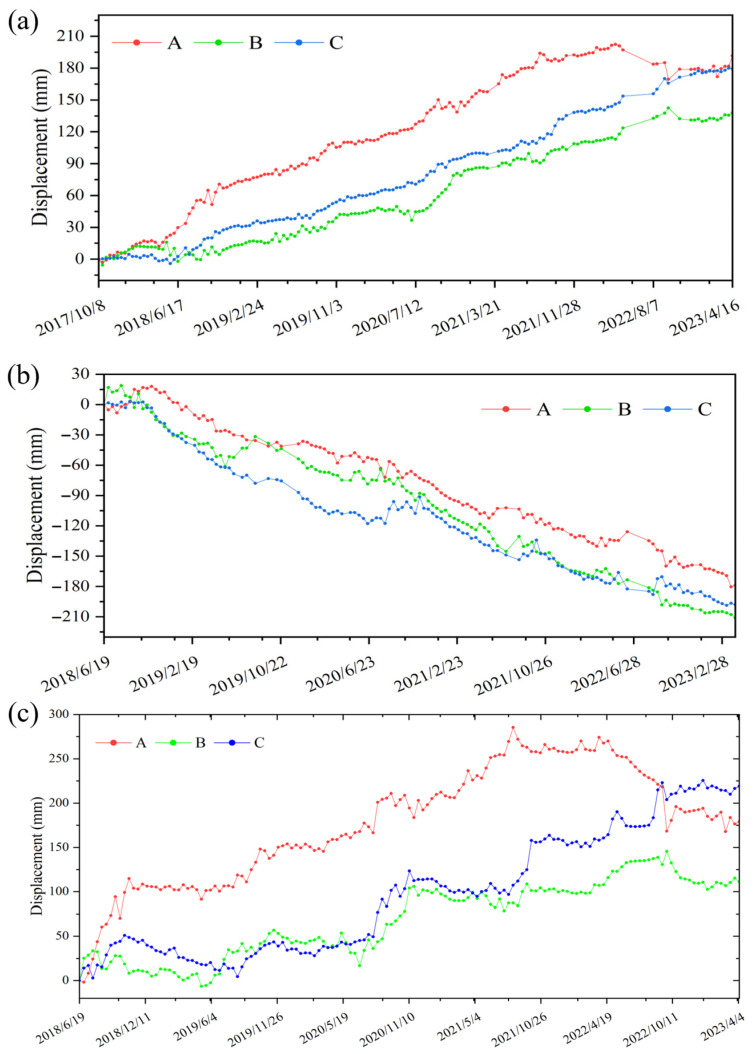
Cumulative deformation results of Zhazishu landslide: (**a**) ascending orbit cumulative deformation, (**b**) descending orbit cumulative deformation, and (**c**) slope-oriented cumulative deformation.

Field verification revealed that the slope exhibits loose soil, sparse surface vegetation, and a small portion has been cultivated as farmland. Abnormal groundwater discharge was observed at the front edge of the slope, and multiple cracks were found on house walls and roads on the middle part of the slope, exhibiting environmental characteristics indicative of landslide development. One feature point was selected from the lower (A), middle (B), and upper (C) parts of the Zhazishu landslide hazard (Figure 6d). The cumulative deformation curves of these feature points (Figure 7a,b) show that cumulative deformation from both ascending and descending monitoring at this landslide hazard point steadily increases over time, with relatively small differences in deformation magnitude across different parts, with point A at the lower part of the landslide showing the largest cumulative deformation.

To address the issue that Sentinel-1A ascending and descending orbit images are not acquired at exactly the same time over the same area, temporal registration must first be performed when jointly processing the cumulative deformation from both orbits to derive slope-oriented deformation. In this study, cubic interpolation was first applied to the descending orbit data to generate 147 consecutive cumulative deformation sequences at 12-day intervals (from 19 June 2018 to 6 April 2023), which were then used as the temporal reference. Subsequently, the ascending orbit data were temporally interpolated and matched to obtain the corresponding cumulative deformation for the ascending orbit.

Subsequently, following the method described in Section 3.1, the slope-oriented deformation rates (Figure 6c) and cumulative displacements at characteristic points (Figure 7c) of the landslide hazard were extracted. The results indicate that the Zhazishu landslide predominantly exhibits downward displacement along the slope aspect (with downward movement defined as positive), and the slope-oriented deformation rates range from −15 mm/a to 50 mm/a. Compared with single-orbit LOS deformation rates, the slope-oriented deformation presents a spatial distribution that more closely aligns with actual topographic characteristics. The slope-oriented deformation rates are highest in the middle–lower part of the landslide, with both the extent and magnitude of deformation increased, thereby more realistically reflecting the actual movement state of the landslide along the slope aspect.

The cumulative displacement trends along the slope aspect at characteristic points A, B, and C of the landslide hazard are generally consistent with the LOS monitoring results from ascending and descending orbits, but with increased overall magnitudes, reaching a maximum cumulative displacement of 270 mm. Characteristic points C and B, located in the upper and middle sections of the landslide, exhibit steadily increasing slope-oriented displacement. Notably, point C consistently shows greater displacement than point B, and this disparity becomes particularly pronounced after November 2021. Notably, although characteristic point A, situated in the lower section of the landslide, exhibited the largest cumulative displacement in the early stage, it began to show a declining trend from July 2021 onward, contrasting with the continuous increases observed at points B and C. This behavior may indicate localized accumulation processes in the lower section, potentially associated with deformation occurring in the mid-upper part of the landslide, although further field evidence is required to confirm this interpretation.

### 4.2. Landslide Deformation Prediction

#### 4.2.1. Landslide Deformation Decomposition and Influencing Factor Selection

**Figure 8:**
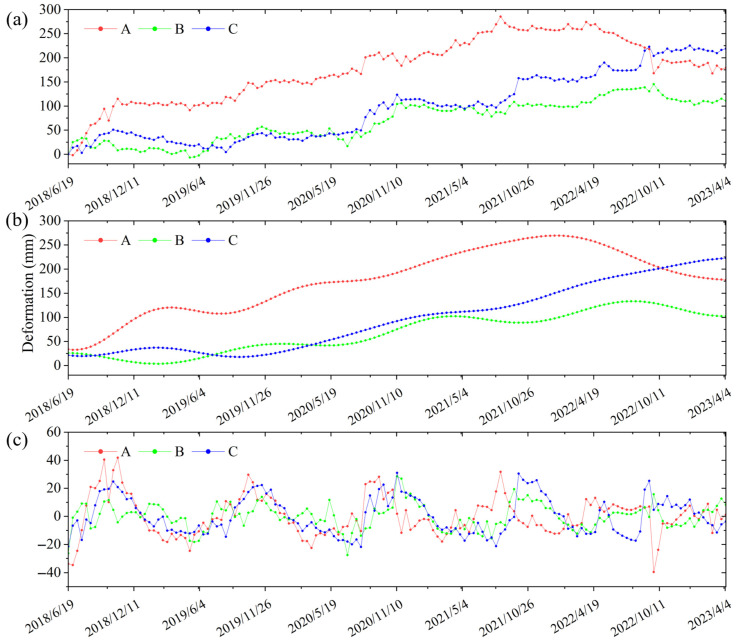
Decomposition results of deformation components: (**a**) total deformation, (**b**) trend component, and (**c**) periodic component.

Based on the obtained slope-oriented cumulative displacement sequences of characteristic points A, B, and C of the Zhazishu landslide hazard (Figure 8a), the wavelet decomposition method was applied. A four-level Daubechies decomposition was selected based on empirical testing to balance noise suppression and signal fidelity. Accordingly, the displacement sequences were decomposed to extract the trend component (Figure 8b) and periodic component (Figure 8c).

**Figure 9:**
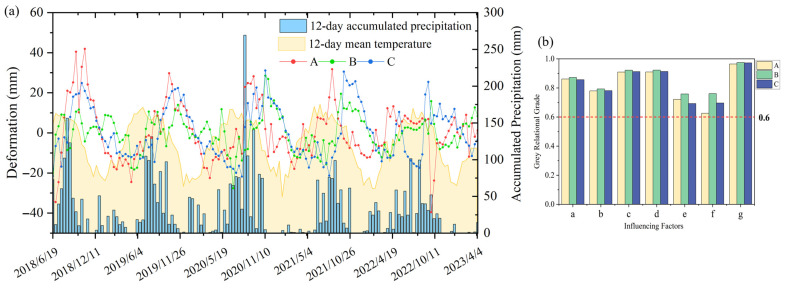
Selection and analysis of influencing factors. (**a**) Relationship between periodic deformation, temperature, and precipitation; (**b**) comparison of gray relational degrees.

Figure 9a clearly demonstrates a significant correlation between the periodic displacement of the landslide hazard and seasonal factors such as average temperature and precipitation, with a certain degree of hysteresis characteristics in the displacement peak responses. Precipitation is the primary external trigger for landslides in Yunnan. It increases pore water pressure within the slope and reduces the shear strength of the soil. Temperature represents seasonal environmental cycles that influence evaporation and the hydrologic state of the slope surface. Furthermore, the landslide’s historical deformation reflects its internal kinematic state and “memory” effect, which significantly constrains future movement. Consequently, this study utilizes periodic displacement at 12-day intervals as the prediction target. Seven sequences were selected as influencing factors (Table 3), and the gray relational grades between each factor and the periodic displacement at the characteristic points of the landslide hazard were subsequently calculated.

It is generally accepted that a gray relational grade greater than 0.6 indicates a strong correlation between the influencing factor and the periodic deformation component [69]. The calculated gray relational grades for all influencing factors listed in Table 3 exceed 0.6 (Figure 9b). Therefore, these seven influencing factors were selected as input features for periodic component prediction.

#### 4.2.2. Deformation Prediction

**Figure 10:**
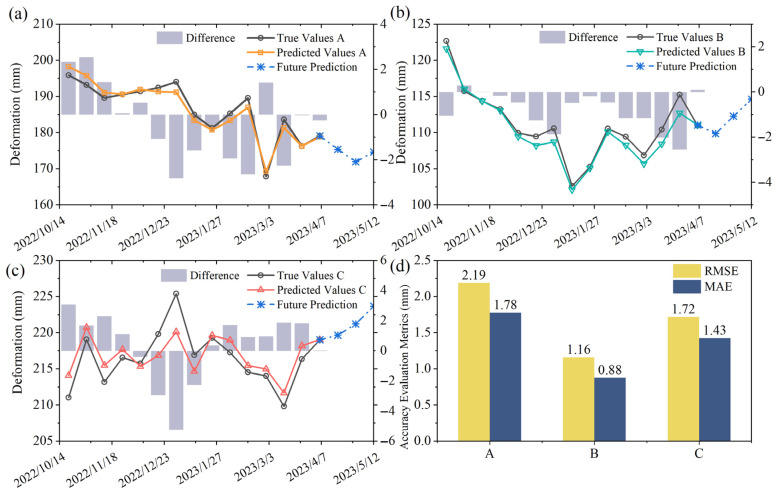
Prediction results of total deformation. (**a**–**c**) Comparison between predicted and measured total deformation at characteristic points A, B, and C; (**d**) comparison of accuracy metrics at each characteristic point.

For the prediction of the trend and periodic components of slope-oriented cumulative displacement at landslide characteristic points, the dataset was divided into training and testing sets with a time step of 3, yielding 129 training samples and 15 testing samples. The testing period spans from 20 October 2022 to 6 April 2023 (approximately 6 months). Separate LSTM models were developed for the two components. The trend component was modeled using a univariate LSTM, while the periodic component was modeled using a multivariate LSTM incorporating selected influencing factors. The Adam optimizer and mean squared error (MSE) loss function were employed. The initial learning rates were set to 0.005 and 0.01 for the trend and periodic components, respectively, with a decay factor of 0.1 applied during training. Model performance was evaluated using root mean square error (RMSE) and mean absolute error (MAE). Each experiment was repeated five times, and the averaged results were used as the final predictions. The total landslide deformation was obtained by summing the predicted trend and periodic components. As shown in Figure 10, which presents the prediction results on the testing set, the predicted slope-oriented deformations at characteristic points A, B, and C of the Zhazishu landslide hazard are in good agreement with the measured values, with RMSE values of 2.19 mm, 1.16 mm, and 1.72 mm, and MAE values of 1.78 mm, 0.88 mm, and 1.43 mm, respectively. These results demonstrate that the proposed model achieves high overall accuracy in predicting the deformation of the Zhazishu landslide hazard. Furthermore, the trained model was used to predict deformation for three future time steps (36 days) beyond the observation period, and the extrapolated results further support the model’s capability for short-term forecasting.

Among the prediction results for the Zhazishu landslide hazard, characteristic point B (middle section) achieved the highest prediction accuracy, while point C (upper section) exhibited relatively lower overall accuracy, primarily due to the more pronounced fluctuations in its periodic displacement peaks. Nevertheless, the model was still able to capture the overall deformation trend with reasonable accuracy. Preliminary analysis suggests that prediction accuracy may be correlated with the magnitude of cumulative displacement, and the prediction accuracy of total displacement closely approximates that of the periodic component. The ranking of prediction accuracy among the characteristic points is consistent with the calculated gray relational grades, indicating that variations in the selected influencing factors exert significant control over landslide deformation. This also demonstrates that the prediction accuracy of the periodic component is a key factor constraining the overall performance of total displacement prediction.

**Figure 11:**
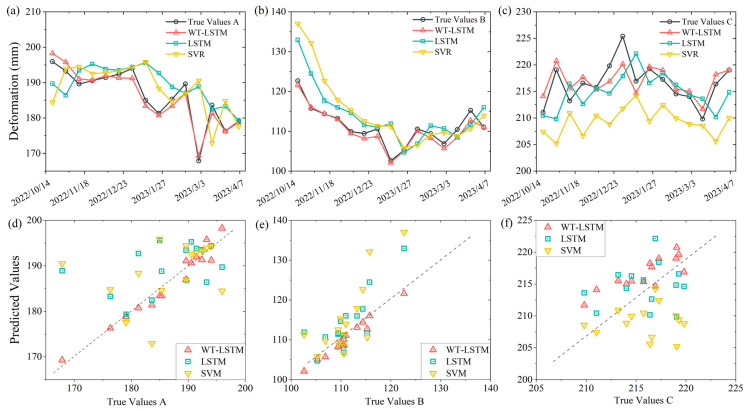
Comparative analysis of prediction results from different models: (**a**–**c**) prediction results of each model for characteristic points A, B, and C, respectively; (**d**–**f**) scatter plots of predicted versus true values for each model at characteristic points A, B, and C, respectively.

To evaluate the credibility and validity of the proposed prediction model, LSTM and SVR models were selected for comparative analysis against the WT-LSTM model in this study. As shown in Figure 11 and Table 4, the prediction results of the LSTM and SVR models are relatively similar, both exhibiting pronounced hysteresis in deformation prediction. In contrast, the stepwise prediction approach combining wavelet transform with the LSTM model effectively mitigates this issue, particularly at characteristic points A and B, where the correlation between predicted and measured values is substantially improved. Although the overall prediction performance at characteristic point C remains suboptimal, the stepwise LSTM prediction method still yields relatively better results. Comparison of prediction performance across the three characteristic points reveals that point B achieves consistently high prediction accuracy across all models, effectively capturing the development trend of cumulative displacement. Points A and C exhibit relatively lower accuracy, suggesting that the adaptability of deformation prediction models may be correlated with the magnitudes of total displacement and periodic component deformation. However, further experimental analysis based on actual conditions is required to validate this inference.

In summary, compared with the other two methods, the prediction approach combining wavelet transform with the LSTM model achieves substantially reduced model accuracy evaluation metrics. This enhanced performance is largely attributed to the selection of influencing factors (including rainfall, temperature, and prior displacement), which provide the model with critical information regarding environmental triggers and the landslide’s historical activity, thereby making the WT-LSTM model more effective in predicting the deformation of the Zhazishu landslide hazard.

## 5. Discussion

This study identified 41 potential landslide hazards in Yangbi County based on ascending and descending time-series InSAR data and extracted the time-series slope-oriented deformation of a typical landslide hazard (the Zhazishu landslide). Furthermore, by integrating wavelet decomposition with the LSTM model, a WT-LSTM prediction model was constructed to achieve reliable landslide deformation prediction. The following discussion addresses the applicability of the methodology and the performance of the prediction model.

### 5.1. Applicability of Ascending and Descending Time-Series InSAR for Landslide Identification in Complex Mountainous Areas

In mountainous regions characterized by rugged terrain and dense vegetation cover, such as Yangbi County, single-orbit InSAR monitoring is particularly susceptible to geometric distortions (e.g., layover and shadowing), resulting in missing or distorted deformation information. This study employed the SBAS-InSAR method to process ascending and descending Sentinel-1A data, effectively preserving interferometric coherence and leveraging the geometric differences in dual-orbit observations to enhance the completeness of landslide hazard detection. The results identified a total of 41 potential landslide hazards within the study area, with 27 detected by ascending orbit data, 22 by descending orbit data, and 8 jointly identified by both orbits. This demonstrates that multi-orbit data fusion helps mitigate the loss of deformation caused by geometric distortions inherent in single LOS observations in steep terrain, and is particularly suitable for alpine canyon regions such as Yunnan. In general, ascending orbit data tend to be more sensitive to east-facing slopes, while descending orbit data are more sensitive to west-facing slopes [31,70]. Therefore, their combined use can help alleviate monitoring blind zones caused by terrain aspect.

**Figure 12:**
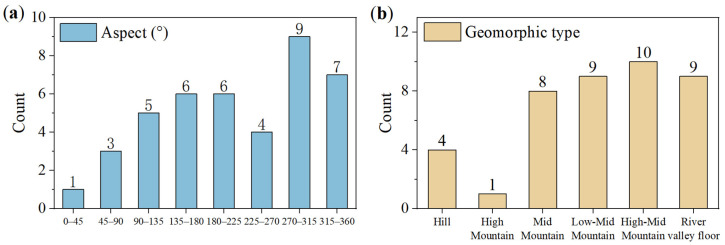
Statistical characteristics of identified landslide hazard sites: (**a**) aspect; (**b**) geomorphic type.

A preliminary statistical analysis of the identified landslide hazard sites has also been conducted, as shown in Figure 12. Notably, the identified landslide hazards exhibit a spatial distribution pattern strongly correlated with topography and geological structures. Although these landslide hazards are dispersedly distributed throughout the county, significant clustering is observed in Cangshan West Town in the northeastern part, along the Yangbi River system of the Lancang River basin, and near the eastern seismic fault zone (involving Fuheng Township, Taiping Township, Wachang Township, among others). This spatial pattern has clear genetic explanations: Cangshan West Town, characterized by steep terrain and intense tectonic activity, provides favorable topographic and material conditions for landslide occurrence; the strong downcutting of the Yangbi River system has reduced bank slope stability, while the areas adjacent to the eastern fault zone, with fragmented rock masses and frequent seismic activity, have further exacerbated slope instability risk. This distribution not only demonstrates the effectiveness of ascending and descending orbit InSAR techniques in regional landslide hazard screening, but also reflects the potential influence of tectonic and geomorphological factors on landslide occurrence. These findings can provide a useful reference for the delineation of priority prevention and control areas.

### 5.2. Interpretation and Early Warning Potential of Slope-Oriented Deformation

Compared with single LOS deformation, slope-oriented deformation can better represent the downslope movement characteristics of landslides. The cumulative slope-oriented displacement obtained in this study through joint inversion of ascending and descending orbit data demonstrated a higher degree of consistency with topography at the Zhazishu landslide, with displacement magnitudes significantly exceeding those of LOS observations. This result confirms that the predominant movement direction of the landslide is downward along the slope surface, which is consistent with landslide physical mechanisms, indicating that slope-oriented displacement can serve as a useful indicator for landslide stability assessment and early warning. However, it should be noted that the reconstruction of slope-oriented displacement relies on the assumption that landslide motion is predominantly parallel to the slope surface. In cases where more complex three-dimensional movements are significant, this assumption may introduce uncertainties into the reconstructed results.

This study also revealed differential deformation trends across different parts of the landslide (upper, middle, and lower sections), which may be related to internal stress adjustment and material migration within the landslide body. This finding provides a useful reference for further investigation of landslide dynamics. However, further validation across multiple sites and integration with additional data sources are needed to fully evaluate its applicability for operational early warning.

### 5.3. Performance and Influencing Factor Analysis of the WT-LSTM Prediction Model

The WT-LSTM model constructed in this study achieved RMSE values ranging from 1.16 to 2.19 mm and MAE values ranging from 0.88 to 1.78 mm in predicting deformation at the three characteristic points of the Zhazishu landslide, demonstrating significantly higher accuracy than both the LSTM and SVR models. This advantage stems not only from the application of the signal decomposition mechanism but also exhibits a close relationship with the influencing factors and spatial heterogeneity of landslide deformation.

Wavelet transform (WT) decomposes the displacement time series into trend and periodic components, enabling the model to specifically characterize deformation components with distinct physical mechanisms: the trend component captures steady deformation of the landslide under long-term processes such as gravity and tectonism, whose monotonic and smooth evolution can be effectively captured using univariate LSTM, while the periodic component, primarily driven by external environmental factors, exhibits fluctuating characteristics. This signal decomposition strategy avoids mutual interference between signals of different origins, thereby enhancing both the structural interpretability and prediction stability of the model. Furthermore, among the seven factors selected through GRA, the periodic displacement showed the highest correlation (>0.9) with short-term (12-day) cumulative precipitation and mean temperature, indicating that summer rainfall serves as a critical external triggering mechanism for landslide deformation in the study area. This finding holds significant implications for advance disaster prevention and preparedness.

The analysis also reveals that the prediction accuracy of total displacement closely approximates that of the periodic component, indicating that the accuracy of periodic component prediction is the key determinant of overall prediction performance. Further observation indicates that the ranking of prediction accuracy among the characteristic points is consistent with the calculated gray relational grades (higher correlation corresponds to relatively better prediction accuracy). This consistency not only validates the significant driving role of the selected influencing factors (e.g., precipitation and temperature) in landslide deformation, but also demonstrates that factor selection via GRA constitutes an effective approach to improving the prediction accuracy of the periodic component.

Comparative analysis of prediction models reveals that although the standalone LSTM and SVR models can roughly track the deformation trend, their predicted curves exhibit pronounced hysteresis and substantial errors, indicating difficulty in simultaneously processing signals mixed with trend and periodic components. By incorporating a prepositive decomposition step, WT-LSTM enables LSTM to focus on learning the evolution patterns of individual components, thereby significantly improving prediction synchrony and accuracy.

In summary, the WT-LSTM model not only achieves high-precision prediction at the overall level, but its prediction performance also aligns with the physical response mechanisms of landslides, further confirming the reliability and applicability of this model for landslide deformation prediction in complex mountainous areas.

### 5.4. Limitations and Future Prospects

This study has preliminarily validated the effectiveness of ascending and descending orbit InSAR combined with the WT-LSTM model for landslide identification and prediction. However, several limitations remain in the systematic study of landslide hazard identification and prediction in complex mountainous areas, which require further improvement in future work. First, although the integration of ascending and descending orbit observations provides complementary information, it remains difficult to obtain highly reliable slope-oriented deformation for landslide hazards identified using single-orbit data. Second, the input variables of the prediction model mainly consider meteorological factors such as precipitation and temperature, while potential triggering factors, including seismic activity, groundwater variations, and human engineering activities, have not yet been incorporated. This may affect the completeness of predictions for landslides characterized by multi-factor coupling mechanisms. In addition, future studies should conduct multi-site experiments covering different landslide types and environmental conditions, and explore multiple predictive models, to further evaluate the general applicability of the proposed method.

## 6. Conclusions

This study focuses on Yangbi County, Dali Prefecture, Yunnan Province, and applies joint ascending and descending time-series InSAR techniques to detect landslide hazards and extract slope-oriented deformation. Wavelet analysis and an LSTM model, combined with selected influencing factors, were further used to predict slope-oriented displacement at characteristic points of a representative landslide. The results provide a case-based reference for landslide monitoring and early warning in complex mountainous areas.

(1) A total of 41 potential landslide hazards were identified using SBAS-InSAR with ascending and descending data, supported by field validation. Among them, 19 were detected only by ascending orbit, 14 only by descending orbit, and 8 by both. The results indicate that multi-orbit observations improve detection completeness in this study area.
(2) For the Zhazishu landslide, a typical landslide hazard, slope-oriented deformation ranges from −15 mm/a to 50 mm/a, with a maximum cumulative displacement of 270 mm. Compared with LOS deformation, the reconstructed results show better consistency with slope geometry and more clearly reflect downslope movement patterns.
(3) Deformation prediction at characteristic points using the WT-LSTM model yields RMSE values of 2.19 mm, 1.16 mm, and 1.72 mm, indicating satisfactory performance within the testing period. Compared with LSTM and SVR, the proposed approach shows improved accuracy, suggesting the benefit of signal decomposition and multi-factor inputs.

This study proposes a technical workflow covering hazard identification, deformation extraction, and time-series prediction, which can provide a reference for the early identification and risk warning of landslide hazards in complex mountainous areas.

## Figures and Tables

**Table 1:** Detailed information on Sentinel-1A data.

Orbit	Path	Frame	Time Range	Number of Scenes	Master Image Date
Ascending	99	1265	8 October 2017 to 16 April 2023	153	4 October 2020
Descending	135	508	19 June 2018 to 6 April 2023	127	6 October 2020

**Table 2:** Statistical Information Table of Landslide Hazards Identified by Ascending and Descending Orbits SBAS-InSAR in Yangbi County.

No.	Longitude	Latitude	Area (km^2^)	Location	Observation Orbit
YB01	99°51′6.92″ E	25°45′23.02″ N	1.10	Wanpo Village, Yangjiang Town	Asc.
YB02	99°58′39.35″ E	25°44′15.61″ N	0.27	Ziyang Village, Yangjiang Town	Asc.
YB03	99°45′20.93″ E	25°40′25.20″ N	0.29	Baiqiao Village, Fuheng Township	Asc.
YB04	99°55′48.32″ E	25°41′14.30″ N	0.10	Baiyang Village, Cangshanxi Town	Asc.
YB05	100°0′37.40″ E	25°39′56.91″ N	0.13	Machang Village, Cangshanxi Town	Asc.
YB06	100°3′10.13″ E	25°38′50.08″ N	0.12	Jinniu Village, Cangshanxi Town	Asc.
YB07	99°44′29.56″ E	25°36′29.52″ N	0.63	Changshou Village, Fuheng Township	Asc.
YB08	99°47′51.57″ E	25°35′43.47″ N	0.15	Pingdi Village, Taiping Township	Asc.
YB09	100°4′21.91″ E	25°36′43.69″ N	0.28	Pingpo Village, Pingpo Town	Asc.
YB10	99°54′6.39″ E	25°31′31.81″ N	0.23	Wayao Village, Shunbi Town	Asc.
YB11	99°48′22.33″ E	25°29′34.82″ N	0.38	Luoshideng Village, Taiping Township	Asc.
YB12	99°51′3.54″ E	25°26′16.68″ N	0.90	Qinghe Village, Longtan Township	Asc.
YB13	99°52′57.66″ E	25°24′15.37″ N	0.21	Longtan Village, Longtan Township	Asc.
YB14	99°57′41.58″ E	25°24′23.16″ N	0.53	Ruhe Village, Wachang Township	Asc.
YB15	99°58′45.39″ E	25°24′32.89″ N	0.19	Ruhe Village, Wachang Township	Asc.
YB16	99°53′14.10″ E	25°21′18.00″ N	0.13	Fuchang Village, Longtan Township	Asc.
YB17	99°59′7.33″ E	25°22′46.85″ N	0.33	Waniwu Village, Wachang Township	Asc.
YB18	99°59′14.82″ E	25°21′46.71″ N	0.26	Waniwu Village, Wachang Township	Asc.
YB19	99°58′56.68″ E	25°20′33.58″ N	0.26	Waniwu Village, Wachang Township	Asc.
YB20	99°39′27.28″ E	25°42′53.21″ N	1.29	Luolimi Village, Fuheng Township	Des.
YB21	99°42′4.66″ E	25°41′31.98″ N	0.24	Fuheng Village, Fuheng Township	Des.
YB22	99°39′37.61″ E	25°39′26.73″ N	0.48	Fuheng Village, Fuheng Township	Des.
YB23	99°57′3.93″ E	25°47′53.26″ N	0.22	Jinzan Village, Yangjiang Town	Des.
YB24	99°59′57.77″ E	25°44′47.89″ N	0.22	Shizhong Village, Cangshanxi Town	Des.
YB25	99°57′9.13″ E	25°42′57.65″ N	0.10	Shizhong Village, Cangshanxi Town	Des.
YB26	100°0′15.57″ E	25°42′14.48″ N	0.29	Meixi Village, Cangshanxi Town	Des.
YB27	100°2′56.26″ E	25°43′8.80″ N	0.28	Meixi Village, Cangshanxi Town	Des.
YB28	100°1′7.11″ E	25°40′13.14″ N	0.22	Machang Village, Cangshanxi Town	Des.
YB29	99°55′9.38″ E	25°39′7.15″ N	0.10	Xiuling Village, Cangshanxi Town	Des.
YB30	99°49′39.99″ E	25°37′20.97″ N	0.76	Pingdi Village, Taiping Township	Des.
YB31	99°48′4.95″ E	25°36′3.90″ N	0.12	Goupi Village, Taiping Township	Des.
YB32	99°50′51.98″ E	25°32′4.79″ N	0.16	Dutian Village, Taiping Township	Des.
YB33	99°56′22.82″ E	25°16′58.85″ N	0.56	Caibai Village, Jijie Township	Des.
YB34	99°53′47.49″ E	25°15′29.63″ N	0.10	Jijie Village, Jijie Township	Asc. and Des.
YB35	99°59′2.62″ E	25°43′23.08″ N	0.38	Shizhong Village, Cangshanxi Town	Asc. and Des.
YB36	99°59′27.28″ E	25°43′31.90″ N	0.33	Shizhong Village, Cangshanxi Town	Asc. and Des.
YB37	100°0′15.84″ E	25°40′47.80″ N	0.34	Machang Village, Cangshanxi Town	Asc. and Des.
YB38	99°59′27.46″ E	25°38′43.88″ N	0.26	Shahe Village, Cangshanxi Town	Asc. and Des.
YB39	99°47′47.22″ E	25°39′56.03″ N	1.10	Goupi Village, Taiping Township	Asc. and Des.
YB40	99°51′4.24″ E	25°35′54.88″ N	0.66	Taiping Village, Taiping Township	Asc. and Des.
YB41	99°54′23.30″ E	25°19′44.11″ N	0.68	Xinzhai Village, Jijie Township	Asc. and Des.

Note: Asc. and Des. denote ascending and descending observation orbits, respectively.

**Table 3:** Gray Relational Degree Calculation Results of Influencing Factors.

Code Number	Influencing Factor	A	B	C
a	12-day accumulated precipitation	0.86	0.87	0.86
b	24-day accumulated precipitation	0.78	0.79	0.78
c	12-day mean temperature	0.91	0.92	0.91
d	24-day mean temperature	0.91	0.92	0.91
e	12-day accumulated deformation	0.72	0.76	0.69
f	24-day accumulated deformation	0.63	0.76	0.70
g	24-day accumulated Periodic Component	0.96	0.97	0.97

**Table 4:** Accuracy evaluation metrics of prediction models.

Predictive Model	A		B		C	
	RMSE/mm	MAE/mm	RMSE/mm	MAE/mm	RMSE/mm	MAE/mm
WT-LSTM	2.19	1.78	1.16	0.88	1.71	1.43
LSTM	7.33	5.34	5.28	4.34	4.50	3.67
SVR	8.32	5.75	7.01	5.22	8.24	7.18

## Data Availability

The datasets generated during and/or analyzed during the current study are available from the corresponding author on reasonable request.
